# Dr. Saunders on Artificial Mineral Waters

**Published:** 1802-12-01

**Authors:** W. Saunders

**Affiliations:** New Broad Street


### *To* Dr. BRADLEY.

DEAR SIR,

MY attention having been particularly engaged for many years paft on the fubject of mineral waters, which I have long confidered as a fertile fource of important remedies, I feel particular pleafure in availing myfelf of your refpectable Journal to direct the attention of medical men on fome improvements lately introduced into this country by Mr. Paul, of Geneva, in the imitation of mineral waters; improvements which he had introduced abroad for many years paft, and which I fhould certainly have taken the opportunity of mentioning in the work which I publifhed about two years ago on that fubject, had they, at that time, reached my knowledge. And I feel the more inclined to take this kind of public notice of the ingenious labours of Mr. Paul, as this gentleman has, in the moft liberal manner, divefted himfelf of any kind of fecret or myftery, with regard to all phyficians, or other competent perfons, who have defired to become acquainted with his inventions and proceffes, and as I am one of thofe to whom he has communicated, without any referve, all that could intereft me in thofe refpects.

Mr. Paul, on his firft introduction in this country, has laid before the public the tranflation of a Report made in the year 1799, to the Inftitute of France, by fome of the moft diftinguifhed chemifts in that country, on his manufacture of artificial mineral waters at Paris. Thefe gentlemen have confidered the eftablifhment in queftion not only as an object of medical and fcientific fcientific inquiry, but alfo as a kind of public concern, and an undertaking which ought be encouraged, f om the national advantages which it is likely to produce.* This report, which is accompanied by the moft favourable certificates from the Faculty of Geneva, and that of Paris, beftows the greateft praife on Mr. Paul, both as a mechanic and chemift, and renders to his eftablifhment the moft authentic juftice. I do not by any means propofe following here the Inftitute of France in their circumftantial examination of Mr. Paul's pneumatic laboratory, and of the mechanical part of his operations. All that I would fay in this refpect is, that the whole of his laboratory, and efpecially his method of impregnating water with gas, have appeared to me fingularly well contrived and executed, and have entirely correfponded with the impreffion which I had received from the reports above mentioned.

In my Treatife on Mineral Waters, I have pretty fully ftated the opinion which I have formed on the utility of that clafs of remedies, and have offered alfo fome conjectures on the mode of their operation. I have attempted to fhow, that the remarkable effects which are obtained from certain fubftances, taken in that diluted form, rather than in a folid fhape, as alfo the apparent difproportion between the minute quantities in which thefe fubftances are taken, and their powerful effects on the animal economy, ought to be attributed partly to their ftate of extreme divifion, partly to the effect of the aqueous vehicle itfelf, and that thofe effects are, in certain cafes, powerfully affifted by an incr afe of temperature.

As to the gafeous waters, and particularly thofe that are ftrongly impregnated with carbonic acid gas, of which Seltzer water is a ftriking inftance, I fhall not repeat here the opinions which I have advanced in the fame work, on the medicinal ufe of thofe waters.† Every body, I believe, is now ready to admit, that in moft dyfpeptic complaints, the portion of gas which efcapes from the liquid immediately on its reaching the ftomach, and is thus applied to that organ in a gafeous form, produces, at leaft, very grateful palliative effects; and as it is generally acknowledged, that the ‡ portion of gafeous acid, which enters the circulation along with its aqueous vehicle, gradually produces on the animal economy other more important, though lefs immediate effects. The univerfal repute which this clafs of waters has gradually acquired in Europe, both as affording a pleafant

* See the Reports, &c. † Treatife on Mineral Waters, p. 232.

‡. Treatife on Mineral Waters, p. 460.

pleasant beverage, and an efficient medicine, and the encouragement which has been given in this and other countries to the artificial preparation of those waters, are, of themselves, strong proof of their beneficial effects; and I feel the greater satisfaction in seeing their utility every day more generally acknowledged, as I was the first who recommended them, at an early period of my medical practice, to the attention of professional men, for the relief of some of the most distressing disorders.*

I have stated at full length in the Treatise, to which I beg leave once more to refer, my opinions on the imitation of mineral springs, and my notions respecting the advantages and disadvantages that may be expected from mineral waters artificially prepared. Mr. Paul has not only distinguished himself by his improvements in the imitation of the natural gaseous springs, but he has also introduced to notice, other gaseous medicinal waters, which are not met with in Nature, and appear to be compositions altogether new and artificial.

Previous to the late improvements in the imitation of mineral waters, this art had for many years been carried to a considerable degree of perfection, by the labours of several natural philosophers, and particularly by those of the illustrious Bergmann. Assisted by accurate analysis, chemists had long since imitated various natural springs, and had even succeeded in impregnating, in some degree, these artificial waters with their gaseous contents, a difficulty which had long appeared unsurmountable.' But Mr. Paul, by long continued labour and experience, and assisted by a careful study of natural philosophy, and of mechanical science, decidedly appears to have arrived at a more perfect imitation of natural springs, than any former chemists; he has, besides, usefully varied and combined these imitations, and has even succeeded in presenting, under a liquid form, certain gaseous substances which Nature never affords in that shape, and which several respectable medical men have already recommended as valuable acquisitions.†

In regard to the natural gaseous waters, and particularly that of Seltzer, Mr. Paul has not only carried their artificial composition, in point of energy and strength, much beyond Nature itself, but he has also introduced a new method of preparing what he calls the *mild* Seltzer water, which has been considered abroad as a real and important improvement. Every one knows the common method of obtaining carbonic acid gas for the purpose of impregnation, which consists on pouring sulphuric acid on

* See a Letter to Dr. Percival in his Medical Essays.

† Reports to the Institute.

on chalk, marble, or any other fort of carbonated lime. This method Mr. Paul employs, for the preparation of his ftrong Seltzer water. But it has been obferved, (and the remark, I believe, has occurred abroad much more frequently than in this country), that thofe waters, when prepared in that degree of ftrength that renders them fo agreeable to the ftomach, are apt to produce in hectic patients and in certain conftitutions extremely irritable, too ftimulating effects. Thefe effects, which are not fo obvious in the natural gafeous waters, have been fuppofed to depend on fome particles of the vitriolic acid being diffolved in the gas and carried along with it into the water. In order to obviate this inconvenience, Mr. Paul has had recourfe to the method of difengaging his gas from chalk by heat alone, and he has found that water prepared with this gas, in the fame degree of impregnation, was milder in its effects, and entirely free from thofe irritating qualities. This idea has had the fulleft approbation of the National Inftitute, and of the Medical Society of Paris. I have had the opportunity of tafting water prepared by that method, and it appeared to me rather lefs agreeable to the palate than the ftrong fort, although perhaps not lefs refembling the natural fpring. With regard to its medicinal qualities, I have not had yet any opportunity of afcertaining them by experience, and this new kind of water has fcarcely yet, I believe, been tried in this country. But admitting it to poffefs thofe advantages in particular cafes, that the French chemifts and the Faculties of Paris and Geneva have afcribed to it, I believe its ufe will be found much lefs general than that of the ftrong fort, particularly in this ifland, where fuch extremely irritable habits are far lefs common, and where a decided preference is likely to be given to the moft agreeable and ftimulating kind.

The Sedlitz water is another fort of artificial mineral water introduced by Mr. Paul in this country. It is another inftance in which Art has confiderably improved the procefs of Nature. This water confifts of vitriolated magnefia, in the proportion of two drachms or even half an ounce to the pint, and is fo powerfully impregnated with carbonic acid, as to render the bitternefs of the falt fcarcely difcoverable. Of this water I have already fome experience, having for fome time been in the habit of prefcribing it as a very pleafant aperient medicine. The Sedlitz water has alfo been tried with fuccefs, in conjunction with a chalibeate, and is likely to prove in this way, a very ufeful tonic purgative, and peculiarly well adapted to difeafes of the liver, fuch as occur both in Europe and in warmer climates, efpecially under habitual coftivenefs, and the diminifhed ed fecretion of bile; the proportion of the magnefia vitriolata may be varied at pleafure.

I have occafionally met with patients who found this water rather more ftrongly impregnated with gas than they could eafily bear; but this can at all times be remedied, fimply by fuffering the water to ftand in the glafs for a few moments before drinking it.

The gafeous alkaline water, commonly called foda water, has long been ufed in this country to a confiderable extent, and has, for many years paft, been prepared in England with great fuccefs. Mr. Paul is fully as happy in this as in other preparations; and he has introduced alfo the gafeous pot-afh waters, to which, in certain cafes, fome practitioners give the preference. Thefe alkaline waters are more extenfively ufed than any other kind of mineral waters, and are certainly, from the large portion of alkali they contain, of great importance in the treatment of feveral diforders. But I cannot help thinking, that a great number of perfons who drink foda water, without any medical interference, and merely on account of the pleafant effect of the gafeous acid on the ftomach, would probably find the Seltzer water more grateful than the foda water, in which the acrid alkaline tafte is more or lefs prevalent, and which may frequently owe the preference which is given to it, to the name having become more familiar.

With refpect to the oxygenated water, and the other kinds of gafeous waters, which are altogether artificial compofitions peculiar to Mr. Paul, I cannot fay I have yet had any opportunity of examining their medicinal effects. But it appears to me, from the authentic Reports of the Faculties of Paris and Geneva, that fome of them, and the oxygenated water in particular, are not unlikely to become ufeful medicines, and that if any advantage in certain cafes may be expected from *oxygenating* the fyftem, an opinion which feveral medical men of character have lately entertained, this would appear to be a much fafer and more rational mode of oxygenation than the means propofed.

I fhall only farther mention another improvement introduced by Mr. Paul, which is that of ufing for his mineral waters, glafs bottles, inftead of the earthen bottles which have hitherto been generally ufed for that purpofe. It is certain that the latter, from their porous texture, and from their being imperfectly glazed, fuffer a quantity of gas to efcape, and even fometimes of the liquid itfelf; whilft, by means of glafs bottles, and with the indifpenfable precaution of laying them on their fides, mineral waters can be preferved for any length of time, without out any lofs of their gafeous contents; and experience has fhown that they can be conveyed, unaltered, to any diftance whatever.

*New Broad Street,*

*Nov.* 5, 1802.

I am, &c.

W. SAUNDERS.

